# Nutritional Properties of *Ogi* Powder and Sensory Perception of *Ogi* Porridge Made From Synthetic Provitamin: A Maize Genotype

**DOI:** 10.3389/fnut.2021.685004

**Published:** 2021-06-25

**Authors:** Omololami Tolulope Akinsola, Emmanuel Oladeji Alamu, Bolanle Omolara Otegbayo, Abebe Menkir, Busie Maziya-Dixon

**Affiliations:** ^1^Department of Food Science and Technology, Bowen University, Iwo, Nigeria; ^2^Food and Nutrition Sciences Laboratory, International Institute of Tropical Agriculture (IITA), Southern Africa Hub, Lusaka, Zambia; ^3^Food and Nutrition Sciences Laboratory, International Institute of Tropical Agriculture (IITA), Ibadan, Nigeria; ^4^Maize Breeding Unit, International Institute of Tropical Agriculture (IITA), Ibadan, Nigeria

**Keywords:** *Ogi* powder, carotenoid retention, biofortification, yellow maize, PVA maize

## Abstract

Provitamin-A maize (PVA) with increased carotenoid content obtained through conventional breeding techniques has been largely successful in sub-Saharan Africa. This resulted in a need to evaluate their susceptibility, retention, and nutritional content during processing into local foods. This study evaluated the chemical, carotenoid composition, and retention of PVA, the phytic acid content in *ogi* powder, and the sensory perception of *ogi* porridge produced traditionally from the three novel PVA maize genotypes (PVA SYN HGAC_0_ Maize 1; PVA SYN HGBC_0_ Maize 2; and PVA SYN HGBC_1_ Maize 3) and one yellow maize variety (control). Chemical composition analyses showed significant differences (*p* < 0.05) in all parameters. The PVA ranged from 5.96 to 8.43 μg/g in Maize 2 and 3 before processing while the true percentage retention after processing into *ogi* powder ranged from 20.25 to 37.54% in Maize 1 and 2, respectively. In addition, there was a reduction in the phytate content of *ogi* powder, and Maize 2 contained the lowest (2.78 mg/g from 4.09 mg/g). Maize 2 genotype had the highest vitamin A contribution; it can meet 18.3% of the vitamin A requirements in children while in adult males and females (>19 years), 6.2 and 7.7%, respectively. Sensory evaluation showed that *the ogi 3 porridge (Maize 3) was the most acceptable*, followed by Maize 2. In conclusion, Maize 2 had the highest PVA, true retention of carotenoid, vitamin A contributions, and the second most acceptable *ogi* porridge with the lowest phytate content.

## Introduction

Maize is an important staple cereal consumed by Africans (1). It is usually produced into various palatable traditional meals such as *ogi* (Nigeria/West Africa), *kenkey* (Ghana), *uji* (Kenya)*, togwa* (Tanzania), *amahewu* (South Africa), *and mawé* (Benin) (2–5). Maize kernel, which is the nutritive part of the plant, contains phytochemicals such as carotenoids, phenolic compounds, and phytosterols (6). It is rich in macronutrients such as starch, fiber, protein, and fat; it also contains substantial amounts of micronutrients such as vitamin B complex, carotenoids, and estimated minerals, i.e., magnesium, zinc, phosphorus, copper, etc. (7). *Ogi* is a popular fermented semisolid food of various colors based on the cereal used for its preparation, ranging between white and yellow from white maize with a slight creamy color and yellow maize (*Zea mays*, yellow variety), and red and white when red or white guinea corn and red or white millet are used. It is commonly consumed as a traditional breakfast and complimentary meal in Nigeria (8–11). The three major tribes in Nigeria refer to the fermented maize gruel (pap) by their different local names; Yoruba (*ogi*), Igbo (*akamu*), and Hausa (*koko*). This meal cuts across the tribes as weaning food. The production of *ogi* involves many unit operations, thus reducing the nutritional quality due to a significant loss of the essential nutrients needed for the body with very little retained (8, 9, 12–14). Furthermore, researchers are making efforts to produce *ogi* of better nutritional quality through the introduction of improved or modified techniques and supplementation of *ogi* with other food materials such as pigeon pea, millet, cowpea, watermelon seed, and African yam bean (15–22).

Vitamin A deficiency is a micronutrient deficiency, posing public health challenge in developing countries, contributing to adverse consequences in children and women of child-bearing age (23–25). The HarvestPlus Challenge Program resulted in the production of biofortified staple crops with maize inclusive due to the prevalence of micronutrient malnutrition in the developing countries due to the consumption of low-density nutrient staple crops (26). This initiative has increased iron, zinc, and provitamin-A maize (PVA) carotenoid levels in staple crops (27). The use of conventional breeding to produce biofortified PVA maize was also stated to have the potential to reduce VAD. The breeding target set by HarvestPlus is 15 μg/g dry weight (DW) of provitamin-A (26, 28, 29).

Countries such as Ghana, Nigeria, and Zambia have been involved in the cultivation of biofortified maize hybrids since 2013, with the Maize Improvement Program of the International Institute of Tropical Agriculture (IITA), Ibadan, being one of the leaders in the development (30–33). Furthermore, the second sustainable development goal (SDG) established by the United Nations in 2015 is Zero Hunger, which is targeted at ending hunger, achieving food security with improved nutrition, and promoting sustainable agriculture (34). Biofortification can, therefore, be considered as a tool to improve nutrition concerning micronutrients (carotenoids).

Carotenoid, a natural pigment present in the hard endosperm of the maize kernel with a small quantity in the germ, is susceptible to degradation during food processing by exposure to heat, light, and air, leading to isomerization, oxidation, and significant loss (2, 35–37). Carotenoids can be divided into two types: provitamin-A (β-cryptoxanthin, β-carotene, and β-carotene) having vitamin A activity, and xanthophylls (lutein and zeaxanthin), which are non-provitamin-A with no vitamin A activity but having anti-oxidative properties. Carotenoids have many health benefits such as modulation of enzymatic activities, activation of gene expression for protein production, anti-oxidative properties enhancing immune system functionality, and reduction of the risk of degenerative diseases (38–41). According to Olson (42), PVA carotenoids are referred to as generic descriptors for all carotenoids that are precursors of vitamin A. This group of compounds is abundant in biofortified varieties of maize compared with the white varieties.

Phytic acid, also known as inostitolhexaphosphate (IP6) or phytate as salt, is reported to be more than 80% in the maize germ and is known to inhibit mineral absorption due to its chelating ability, leading to the formation of insoluble metal-phytate complexes (43). Phytic acid can be broken down in many ways: endogenously by enzymes (e.g., phytase in maize), exogenous enzymes (biotechnologically produced phytase), intestinal mucosal phytase, and microbial phytase (e.g., bacteria, fungi, and yeast) as a result of fermentation, a unit operation in food processing (44, 45). Thus, it reduces the bioavailability and digestibility of proteins and carbohydrates. However, it is the storage form of phosphorus, which accounts for 50–80% of total phosphorus (46–48). Therefore, the fermentation process is essential for reducing phytic acid, as observed in various researches (21, 49, 50). The determination of phytic acid in this maize pipeline is necessary due to farming practices such as the type and quantity of fertilizer that could cause elevated levels. This is because phytate might inhibit the absorption of minerals and nutrients necessary for growth in children. O*gi* porridge prepared from *ogi* powder is a portion of typical complementary food for weaning in Nigeria.

For determining the effect of biofortification on health and nutritive well-being, the estimation and quantification of the micronutrient in the processed products from biofortified crops are essential. This has prompted many studies in different parts of the world to analyze the retention of PVA after processing (51–54). Due to the continuous development of these new PVA maize genotypes, processing of different traditional products and evaluating their carotenoid retention before the advancement for their release to consumers are essential. Thus, this study evaluates the chemical composition, carotenoid composition, and retention of PVA in *ogi* powder. In addition, the evaluation of phytic acid content in *ogi* powder and the sensory perception of *ogi* porridge produced traditionally from the new maize lines of three new PVA maize and one yellow maize variety (control) were executed.

## Materials and Methods

### Materials

Four maize genotypes which were selected from six maize genotypes based on the levels of provitamin-A (planted in three replications and tow seasons), were obtained from the research farms of the Maize Improvement Program at the IITA, Ibadan, Nigeria. The maize genotypes were obtained through conventional breeding techniques based on the planting cycle and their parent lineage were named as follows: PVA SYN HGAC_0_ (provitamin-A biofortified maize HGA cycle zero), PVA SYN HGBC_0_ (provitamin-A biofortified maize HGB cycle zero), PVA SYN HGBC_1_ (provitamin-A biofortified maize HGB cycle one), and DT STR SYN2-Y (yellow landrace maize, used as the control). The three PVA maize genotypes (PVA SYN HGAC_0_, PVA SYN HGBC_0_, and PVA SYN HGBC_1_) were labeled Maize 1, 2, and 3, respectively, while Maize 4, the yellow landrace maize, was used as the control (DT STR SYN2-Y).

### Methods

#### Product Preparation

##### Maize Flour

About 1 kg of maize grain of each variety was sorted and cleaned, and 100 g of each kernel was milled individually to 0.5 mm particle size by using a laboratory mill (3,100 Perten Instrument). The milled samples were left to cool in a dark room, packaged in airtight high-density polyethylene whirl-pak, put in a white envelope to prevent exposure to light, and kept in a −80°C freezer before laboratory analysis. All analyses were done within 48 h of sample preparation to prevent the carotenoid degradation by light and oxygen.

##### Processing Maize Into *ogi* Powder

**Figure 1:**
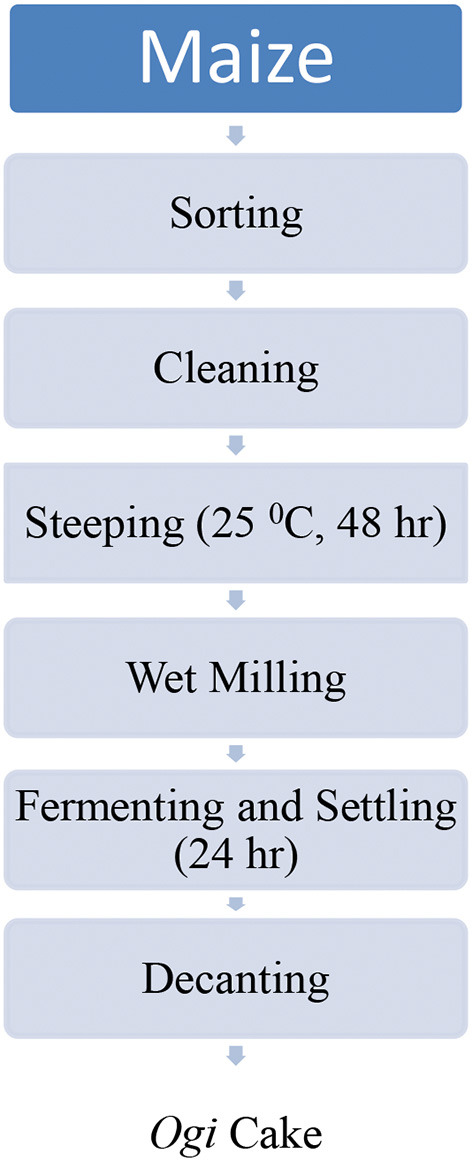
Flow chart for the traditional production of *ogi* cake in Nigeria (55).

**Figure 2:**
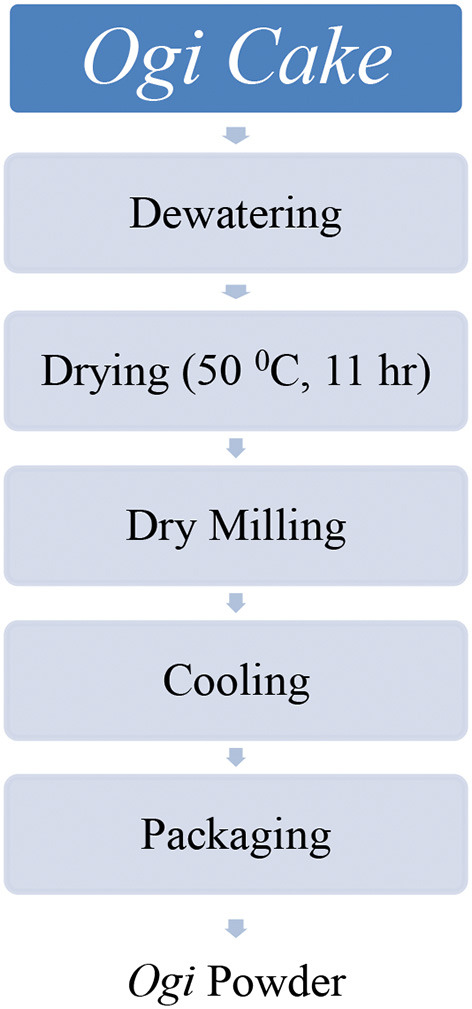
Flow chart for *ogi* powder production (53, 54).

**Plate 1:**
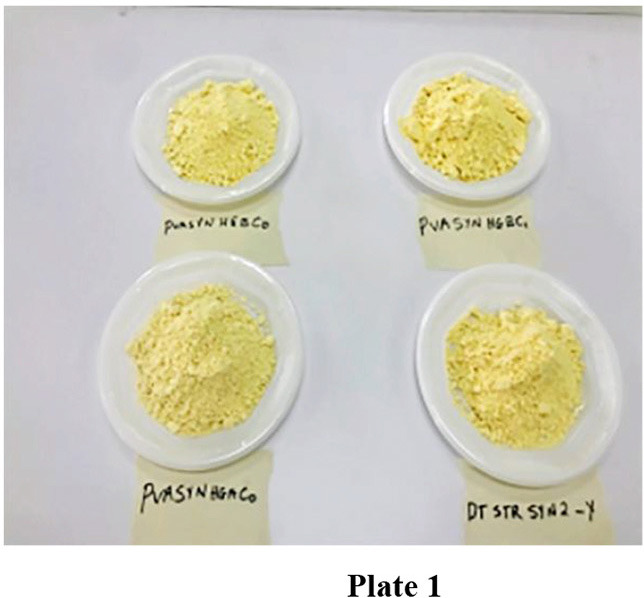
*Ogi* powder from the three provitamin-A biofortified maize genotypes and yellow maize (control).

Figures 1, 2 show the flow chart for *ogi* powder production. Two phases were used: (1) the production of *ogi* cake and (2) processing the cake into powder. First, the traditional processing method was followed; the maize grains were removed from the maize cob, sorted, and about 2 kg were cleaned and steeped in clean water at room temperature for 48 h, after which the water was decanted, and the fermented grains were washed thrice. Next, the fermented grains were wet-milled with the aid of an attrition mill. The slurry was passed through a muslin cloth, the chaff removed, and the filtrate allowed to settle for 24 h to form *ogi* slurry (55). The *ogi* slurry was poured into the muslin cloth and pressed to remove water to obtain the *ogi* cake. The *ogi* cake was placed on a clean, flat stainless steel tray, spread evenly, and dried in a cabinet dryer at 50°C for 11 h. The dried *ogi* cake was then dry milled by using a stainless steel blender (USHA Stainless steel mixer grinder, Model-MG 2053N, USHA International, 15, Institutional Area, Sector 32, Gurugram, Haryana 122018, India) and left to cool before packing in an airtight whirl-pak (53, 54). Plate 1 shows *ogi* powder from the three provitamin-A biofortified maize genotypes and the yellow maize (control).

##### *Ogi* Porridge

**Plate 2:**
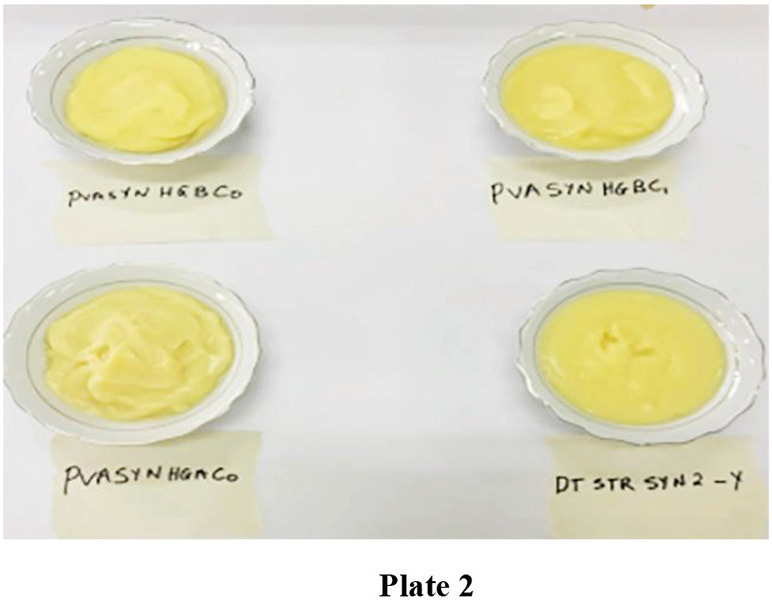
*Ogi* porridge from the three provitamin-A biofortified maize genotypes and yellow maize (control).

The *ogi* powder was prepared into porridge by using the reconstitution method (56) with a bit of modification. *Ogi* powder (200 g) was reconstituted with 1,400 ml of tap water, homogenized, and the slurry was heated with constant stirring for 3 min on a gas cooker. The porridge samples were kept separately in thermos flasks for sensory evaluation and dished out at intervals to 20 untrained panelists conversant with *ogi* consumption. Plate 2 shows *ogi* porridge from the three PVA biofortified maize genotypes and yellow maize (control).

### Determination of Provitamin-A Using High-Performance Liquid Chromatography

The method of Howe and Tanumihardjo (28) was adapted for carotenoid analysis. The quantities of β-cryptoxanthin, β-carotene, and trans with cis isomers of β-carotene were extracted as described by Alamu et al. (57). PVA is calculated as the sum of β-carotene (13-cis-β-carotene, 9-cis-β-carotene, and trans isomers) and half of the addition of β-cryptoxanthin and α-carotene (57).

### Retention of Carotenoids

True retention of carotenoids were determined in percentage by dividing the multiplication value of nutrient content per gram of the processed food (*ogi* powder) and the weight after processing by the multiplication value of nutrient content per gram of raw food (maize flour) and the weight of food before processing (58). The following equation was used:

(1) $$\text{True retention }(\%)=\frac{\text{Nutrient content per g of processed food }\times \text{ weight after processing}}{\text{Nutrient content per g of raw food }\times \text{ weight of food before processing}}\times 100$$

### Contribution to Vitamin A Intake

To calculate the possible contribution of the selected varieties to vitamin A intake when further processed into *ogi* maize gruel/pap, a conversion factor reported by Fadupin (59) was used to calculate the cooked yield if the powder was prepared into pap (which is the usual method of consumption). The serving size reported by Sanusi and Olurin (60) was used to estimate portion size. A bioconversion of 3.2 μg to one retinol activity equivalents (RAE) was then applied (61).

### Chemical Composition Determination

Samples were analyzed for moisture, ash, crude protein, crude fat, and crude fiber by the methods of AOAC (62) and AACC (63). Carbohydrate content were determined by difference. All analyses were done three times.

### Phytate Determination

The method of Wheeler and Ferrel (64) was used. Phytate was extracted with 25 ml of 3% trichloroacetic acid (TCA) after shaking with a shaker for 1 h and centrifuging for 15 min at 1000 rpm. A total of 10 ml of the supernatant was transferred into a centrifuge tube, precipitated by using 4 ml of FeCl_3_ solution, and heated in a water bath at 60–70°C for 45 min. The precipitate was centrifuged for 15 min at 3,500 rpm and then decanted. Washing of the precipitate was done two times by using 25 ml of 3% TCA followed by heating in a water bath for 10 min at 60–70°C and centrifuging at 3,500 rpm. After it was washed one time in distilled water before adding 5 ml of single distilled water and 3 ml of 1.5N NaOH, it was mixed by using a vortex mixer. The mixture was made up to 30 ml with distilled water and heated in a water bath for 30 min at 60–70°C. The mixture was filtered hot into a conical flask with Whatman number two filter paper and the precipitate was washed with 70 ml of hot distilled water, after which the filtrate was discarded. The precipitate was put in 100 ml flasks and dissolved with 40 ml hot 3.2N HNO_3_ (nitric acid). The filter paper was washed with several portions of distilled water, cooled to room temperature, and diluted to volume. An aliquot of 5 ml was put in a 100 ml volumetric flask and diluted with ~70 ml of single distilled water. KSCN (20 ml) was added to the volume, and the absorbance was read at 470 nm wavelength on the spectrophotometer (Thermo Scientific Genesys 10S UV-VIS). A reagent blank was run with the samples. The phytate content (mg/g) was calculated by the subtraction of absorbance from intercept multiplied by dilution factor, the ratio of total solution to the quantity taken, the ratio of Fe to phytic phosphorus, and the ratio of phytic phosphorus divided by sample weight and gradient.

### Sensory Evaluation

Sensory evaluation was carried out on the *ogi* porridge from PVA maize genotypes and the yellow maize (control). The preference test based on the degree of likeness was used. In the test, 20 untrained panelists comprising male and female staff of IITA, Ibadan, Department of Food Nutrition Science Laboratory (FNSL), who were conversant and are frequent consumers of *ogi* porridge, were used. The sensory evaluation questionnaires were based on a one to nine hedonic scales, where one represents “dislike extremely” and nine represents “like extremely” for all the quality attributes employed (65). The *ogi* porridge samples were evaluated for color, taste, texture (mouth feel), appearance, flavor, and overall acceptability in the questionnaires. The four *ogi* porridge samples were served at intervals from separate Thermo flasks into four white plastic cups three times identified with a three-digit code. White plastic spoons were placed for use and clean water for mouth rinsing between the evaluation to avoid a carry-over effect. The panelists were kept in well-illuminated, separate booths during the evaluation to prevent bias and encourage individual judgment.

### Statistical Analysis

All data were statistically analyzed by using SPSS version 20. ANOVA was used to establish significant differences at *p* < 0.05. The mean values were separated by using Duncan's multiple range tests (DMRT). The study used four maize genotypes in two planting seasons with two replications in the field, and three replications were carried out in the laboratory.

## Results and Discussion

Tables 1, 2 show the carotenoid profile of the maize genotypes before (maize flour) and after processing into *ogi* powder. There were significant differences in the carotenoid profile among the maize samples. The carotenoid profile of maize flour showed that it ranged from 18.89 to 21.42 μg/g for total xanthophylls, 1.92–4.06 μg/g for β-cryptoxanthin, 0.62–1.10 μg/g for β-carotene, 0.85–1.31 μg/g for 13-cis-β-carotene, 2.10–3.25 μg/g for trans-β-carotene, 0.88–1.67 μg/g for 9-cis-β-carotene, 3.83–6.16 μg/g for total β-carotene, and 5.96–8.43 μg/g for provitamin-A. For *ogi* powder, total xanthophylls were 5.89–10.31 μg/g, β-cryptoxanthin 1.01–1.74 μg/g, β-carotene 0.27–0.62 μg/g, 13-cis-β-carotene 0.35–0.54 μg/g, trans-β-carotene 0.81–1.19 μg/g, 9-cis-β-carotene 0.41–0.69 μg/g, total β-carotene 1.57–2.42 μg/g, and provitamin-A 2.27–3.10 μg/g. The presence and quantity of carotenoid isomers detected by the HPLC analyses such as 13-cis-β-carotene, 9-cis-β-carotene, and 15-cis-β-carotene in foods have been attributed to food processing, varietal difference, sample storage, and handling (66).

**Table 1:** Carotenoid profile of maize grains before processing [dry weight (DW) basis].

**Genotypes**	**^+^Total xanthophylls (μg/g)**	**β-cryptoxanthin(μg/g)**	**α-carotene (μg/g)**	**13-cis- β-carotene (μg/g)**	**Trans- β-carotene(μg/g)**	**9-cis- β-carotene (μg/g)**	**Totalβ-carotene(μg/g)**	**Provitamin-A (μg/g)**
**Maize 1**	20.98 ± 0.15^b^	3.19 ± 0.01^b^	0.82 ± 0.01^b^	1.31 ± 0.00^d^	3.21 ± 0.01^c^	1.61 ± 0.01^c^	6.13 ± 0.02^c^	8.13 ± 0.02^c^
**Maize 2**	18.89 ± 0.21^a^	1.94 ± 0.02^a^	0.62 ± 0.01^a^	0.93 ± 0.20^b^	2.42 ± 0.04^b^	1.34 ± 0.02^b^	4.69 ± 0.08^b^	5.96 ± 0.08^a^
**Maize 3**	22.89 ± 0.34^d^	3.54 ± 0.05^c^	0.97 ± 0.05^c^	1.27 ± 0.03^c^	3.25 ± 0.08^c^	1.67 ± 0.06^d^	6.18 ± 0.12^c^	8.43 ± 0.12^d^
**Maize 4**	21.42 ± 0.01^c^	4.06 ± 0.00^d^	1.10 ± 0.00^d^	0.85 ± 0.00^a^	2.10 ± 0.00^a^	0.88 ± 0.00^a^	3.83 ± 0.00^a^	6.41 ± 0.00^b^

*Values are mean ± SD of triplet determinations*.

+ *Values with different superscripts along the column are significantly different from one another (p ≥ 0.05)*.

**Table 2:** Carotenoid profile of *ogi* powder (DW basis).

**Genotypes**	**^+^Total xanthophylls (μg/g)**	**β-cryptoxanthin(μg/g)**	**α-carotene (μg/g)**	**13-cis -β-carotene (μg/g)**	**Trans- β-carotene (μg/g)**	**9-cis- β-carotene (μg/g)**	**Total β-carotene (μg/g)**	**Provitamin-A (μg/g)**
**Maize 1**	7.58 ± 0.07^b^	1.14 ± 0.08^a^	0.27 ± 0.02^a^	0.35 ± 0.01^a^	0.81 ± 0.00^a^	0.41 ± 0.01^a^	1.57 ± 0.03^a^	2.27 ± 0.06^a^
**Maize 2**	10.14 ± 0.35^c^	1.01 ± 0.05^a^	0.37 ± 0.01^ab^	0.54 ± 0.04^a^	1.19 ± 0.04^c^	0.69 ± 0.00^c^	2.42 ± 0.07^c^	3.10 ± 0.09^c^
**Maize 3**	5.89 ± 0.39^a^	1.58 ± 0.10^b^	0.54 ± 0.02^bc^	0.45 ± 0.02^b^	0.85 ± 0.09^a^	0.51 ± 0.05^b^	1.80 ± 0.11^b^	2.87 ± 0.18^b^
**Maize 4**	10.31 ± 0.58^c^	1.74 ± 0.13^b^	0.62 ± 0.20^d^	0.38 ± 0.03^b^	0.97 ± 0.03^b^	0.39 ± 0.02^a^	1.74 ± 0.07^b^	2.91 ± 0.03^b^

*Values are mean ± SD of triplet determination*.

+ *Values with different superscripts along the column are significantly different from one another (p ≥ 0.05)*.

Total xanthophyll is the summation of lutein and zeaxanthin; it is reported to possibly possess antimutagenic and anticarcinogenic properties with no vitamin A activity. It acts as an antioxidant (67–70). The range of total xanthophylls before processing was 18.89–22.89 μg/g; Maize 3 had the highest value (22.89 μg/g) and Maize 2 had the lowest (18.89 μg/g). Among the *ogi* powder samples, Maize 3 had the lowest content of 5.89 μg/g from the initial content of 22.89 μg/g. In contrast, Maize 4 *ogi* powder (control) had the highest content, 10.31 μg/g of total xanthophylls (Table 2). However, Maize 2 *ogi* powder contained the highest total xanthophyll (10.13 μg/g). The result showed that the genotype with the lowest content before processing (Maize 2) had the highest content after processing. This implies that Maize 2 *ogi* powder can provide a good source of antioxidants during consumption. However, (53, 54) reported a similar result for total xanthophylls at 0 weeks of storage for the yellow-seeded maize *ogi* powder.

Maize 4 *ogi* powder had the highest α-carotene content of 0.62 ± 0.20 μg/g from 1.10 ± 0.00 μg/g while Maize 1 *ogi* powder had the lowest (0.27 ± 0.02 μg/g from 0.82 ± 0.01 μg/g). Table 2 shows that Maize 2 *ogi* powder contained more β-carotene (0.54 ± 0.02 μg/g) than the other biofortified PVA maize genotypes. β-carotene is a precursor of vitamin A, providing PVA activity, although it is not as potent as β-carotene because it provides only one molecule of vitamin A (71, 72). Trans-β-carotene in maize flour ranged from 2.10 to 3.25 μg/g; after processing into *ogi* powder, it ranged from 0.97 to 1.19 μg/g. Maize 2 had the lowest β-carotene among the biofortified PVA maize genotypes before processing. However, after processing, it contained the highest value in the *ogi* powder with 1.19 ± 0.04 μg/g from the initial content of 2.42 ± 0.04 μg/g while Maize 1 *ogi* powder had the lowest content (0.81 ± 0.00 μg/g). This implies that Maize 2 will possess the highest PVA activity and high conversion to vitamin A. β-carotene occurs in the *trans* form in whole/intact grains. However, it is highly unstable, especially in dehydrated foods, and it is converted to *cis* forms in the presence of light, oxygen, moisture, and high temperature (36). It is a precursor for vitamin A and the most potent vitamin A active form of carotenoid because it can provide two retinol molecules (71, 72).

Maize 2 had the highest 13-cis-β-carotene content of 0.54 ± 0.04 μg/g in the *ogi* powder from the initial value in maize flour (0.93 ± 0.20 μg/g) while Maize 1 had the lowest value (0.35 ± 0.01 μg/g) in the *ogi* powder. The value for Maize 4 (control) was 0.38 ± 0.03 μg/g. Maize 2 also had the highest 9-cis-β-carotene content of 0.69 ± 0.00 μg/g in the *ogi* powder from 1.34 ± 0.02 μg/g while Maize 4 had the lowest value (0.39 ± 0.02 μg/g). Maize 2 experienced isomerization (to 13-cis-β-carotene and 9-cis-β-carotene) more than other maize genotypes after processing to *ogi* powder. The occurrence of isomerization from trans-β-carotene, which is highly unstable to cis-β-carotene, was taken place during the dry milling and other unit operations in the processing method. It occurs the most when heating at atmospheric pressure and temperature lower than 100°C. 13-cis isomer is reported to be formed at higher rates than 9-cis isomer (36, 53, 54, 73); however, from the result, it can be observed that there was more conversion of trans-β-carotene to 9-cis-β-carotene, which could be due to the many unit operations in the traditional processing method.

Total β-carotene ranged from 3.83 to 6.18 μg/g before processing, and after processing, it ranged from 1.57 to 2.42 μg/g, and they are significantly different from one another. It was observed from the result that Maize 2 contained the lowest level of total β-carotene among the biofortified PVA maize genotypes before processing (4.69 ± 0.08 μg/g) into *ogi* powder with a retained composition of 2.42 ± 0.07 μg/g. At the same time, Maize 1 had the lowest value (1.57 ± 0.03 μg/g) in the *ogi* powder from 6.13 ± 0.02 μg/g, which was one of the highest compositions before processing. The value for the Maize 4 (control) was 1.74 ± 0.07 μg/g. Total β-carotene is the summation of trans-β-carotene, 13-cis isomer, and 9-cis isomer. From the result, Maize 2 could retain total β-carotene and thus a higher bioconversion to vitamin A.

The PVA content before processing ranged from 5.96 to 8.43 μg/g while after processing into *ogi* powder, it ranged from 2.27 to 3.10 μg/g. The result showed that PVA SYN HGBC_0_ had the lowest PVA content before processing and that it retained the highest after processing to *ogi* powder. Also, Maize 1 had the highest PVA content before processing while after processing it retained the lowest content (2.27 ± 0.06 μg/g). Maize 2 had the highest PVA value of 3.10 ± 0.09 μg/g from the initial value of 5.96 ± 0.08 μg/g while Maize 1 had the lowest value, 2.27 ± 0.06 μg/g from 8.13 ± 0.02 μg/g and Maize 4 (control) had 2.91 ± 0.03 μg/g (Table 2). Thus, Maize 2 had higher PVA among the *ogi* powder samples irrespective of the effect of processing. However, the amount in μg/g was lower after processing than the HarvestPlus target value of 15 μg/g (DW) (74).

Carotenoids in maize kernels are found mainly in the hard endosperm with a little in the germ and pericarp (37, 75, 76); however, they undergo degradation during food processing. This study revealed that unit operations (wet milling, wet sieving, fermenting and settling, drying at 50°C, and dry milling) in the traditional processing method brought about the exposure of the food product matrix to heat, light, and metal, which resulted in oxidative degradation and isomerization leading to a reduction in carotenoid level (27, 77). Furthermore, the Maize 2 genotype had the highest PVA content in microgram/gram (μg/g) after processing compared with the content being the lowest before processing.

### Carotenoid True Retention Profile

Table 3 presents the true retention of total xanthophylls, total β-carotene, and PVA in the *ogi* powder. For determining the usefulness of biofortification to health and nutritive well-being, the estimation and quantification of the micronutrients are essential in the processed product from biofortified crops. True retention is a way to determine the effectiveness of micronutrient biofortification in improving the nutritional content and sufficient availability after conventional processing and food preparation. The study revealed that Maize 2 (PVA SYN HGBC_0_) had the highest retention value for total xanthophylls, total β-carotene, and provitamin-A in the true retention profile. It also showed that the *ogi* powder from Maize 2 contains more PVA than the *ogi* powder samples from other biofortified provitamin-A genotypes and the yellow maize control. The processing of these maize varieties into *ogi* powder had numerous unit operations (wet milling, wet sieving, fermenting, and settling, drying at 50°C, and dry milling), which brought about a reduction in the PVA level. The carotenoids were lost due to chemical degradation, such as isomerization, oxidation, physical loss through the leaking of soluble solids into the water, increment in surface area, temperature, light, and oxygen (77, 78). The retention results presented here were compared with those of a previous study on *ogi* powder (53, 54).

**Table 3:** True retention of total xanthophylls, total β-carotene, and provitamin-A maize (PVA) in *ogi* powder (DW basis).

**Genotypes**	**^+^Total xanthophylls (%)**	**Total β-carotene (%)**	**Provitamin-A (%)**
**Maize 1**	26.25 ± 0.19^b^	18.57 ± 0.24^a^	20.25 ± 0.12^a^
**Maize 2**	38.64 ± 0.96^d^	37.13 ± 0.42^d^	37.54 ± 0.49^d^
**Maize 3**	20.86 ± 1.53^a^	23.63 ± 1.90^b^	27.53 ± 2.04^b^
**Maize 4**	35.38 ± 1.99^c^	33.20 ± 1.28^c^	33.23 ± 0.34^c^

*Values are mean ± SD of triplet determinations*.

+ *Values with different superscripts along the column are significantly different from one another (p ≥ 0.05)*.

Compared with the findings on boiled maize presented by Alamu et al. (31), the retention of β-carotene was much lower. The reduction in total xanthophyll content from the result could be due to heat-treatment/heat generated during processing (wet milling and drying). This is contrary to the findings of other researchers who observed a slight increase in total xanthophyll content (52, 79). The result shows that carotenoids in biofortified PVA maize will reduce with an increase in the processing steps, especially the unit operations, which involve heat, extended exposure to light, and physical friction. This depleting impact of processing on PVA in maize was also confirmed by Ortiz et al. (80), where the impact of steeping (fermentation) was probed in maize genotypes. It was also observed that both processing and genotypes influence carotenoid retention. This means that the genotypes with the higher PVA before processing may not necessarily retain the most PVA after processing, as seen in this study.

### Chemical Composition of *Ogi* Powder

Table 4 presents the chemical composition of the *ogi* powder from biofortified PVA maize genotype and yellow maize. The moisture content ranged from 6.73% to 8.77%, crude protein 7.82–8.72%, crude fat 3.89–6.03%, crude fiber 0.37–0.85%, ash 0.49–0.52%, and carbohydrate 77.47–79.51%. There were significant differences in all the parameters at a *p*-value of 0.05 (Table 4).

**Table 4:** Chemical composition of *ogi* powder from PVA maize genotype and yellow landrace (control).

**Genotypes**	**^+^Moisture (%)**	**Protein (%)**	**Crude fiber (%)**	**Crude fat (%)**	**Ash (%)**	**Carbohydrate (%)**
**Maize 1**	8.77 ± 0.04^d^	8.72 ± 0.26^b^	0.40 ± 0.04^a^	3.89 ± 0.05^a^	0.48 ± 0.00^ab^	77.72 ± 0.13^b^
**Maize 2**	7.71 ± 0.06^b^	7.82 ± 0.03^a^	0.50 ± 0.02^b^	6.03 ± 0.04^d^	0.49 ± 0.01^a^	77.47 ± 0.03^a^
**Maize 3**	8.27 ± 0.02^c^	7.84 ± 0.08^a^	0.85 ± 0.01^c^	4.22 ± 0.07^b^	0.52 ± 0.01^c^	78.31 ± 0.13^c^
**Maize 4 (control)**	6.73 ± 0.04^a^	7.92 ± 0.01^a^	0.37 ± 0.08^a^	4.99 ± 0.10^c^	0.49 ± 0.01^a^	79.51 ± 0.05^c^

*Values are mean ± SD of triplet determinations*.

+ *Values with different superscript along the column are significantly different from one another (p ≥ 0.05)*.

Maize 1 had the highest moisture content (8.77%) while the content of Maize 2 was the lowest among the PVA genotypes (7.71%); the control obtained the lowest value (6.73%). The observed result is comparable with the result of previous studies (17, 81–84). All the *ogi* powder samples had low moisture content values, which implies that there will be low water activity, which will reduce the growth of bacteria, yeast, and mold. In addition, the low moisture content of the *ogi* powder encourages a longer shelf life, storage ability, and stability of products.

The crude protein content of the different maize genotypes varied from 7.82 to 8.72% (Table 4). The significantly highest value was observed in Maize 1 (8.72%) while Maize 2 had the lowest (7.82%) compared with Maize 4 (control) (7.92%). The result is similar to that reported by Ufot and Winifred (85) and Charles et al. (82) and was higher than that reported by Esther et al. (17) and Eke-Ejiofor and Belaya (2017) while the report from Oludumila and Enujiugha (83) was higher than that observed in this study. The differences observed among the *ogi* powder samples can be attributed to the effect of processing on genetic properties resulting in the denaturation of proteins. In addition, most of the proteins are located in the testa and germ and are exposed by wet milling, resulting in denaturation during processing.

The crude fat content of the samples ranged from 3.89 to 6.03%, with *ogi* powder from Maize 2 having the highest value (6.03%) and Maize 1 having the lowest (3.89%). The value for the yellow landrace was 4.99%. This result is similar to that observed by previous studies (82, 85) (Eke-Ejiofor and Belaya, 2017) and higher than that reported by Esther et al. (17) and Oludumila and Enujiugha (83). The significant differences can be attributed to the breakdown and transformation of the maize matrix component during processing, result in exposure, and the reduction of fat in the maize germ. However, the high fat and provitamin-A contents of Maize 2 could be possible because carotenoids are fat-soluble and could mean that the higher the fat content of the *ogi* powder, the higher its provitamin-A value.

Maize 3 contained the highest crude fiber (0.85%) while the control (Maize 4) had the lowest value (0.37%). The result is similar to that reported by previous authors (85) (Eke-Ejiofor and Belaya, 2017). The significant differences (*p* < 0.05) observed among the *ogi* powder can be due to processing, particularly wet sieving, a unit operation that reduced the fiber content during the removal of the bran.

The ash content of Maize 1, 2, and 4 was not significantly different (Table 4). Maize 3 had the highest ash content (0.52%). The results obtained in this study are lower than those reported by previous authors (17, 81, 82, 85). The ash content of food is important because it gives an idea of the mineral elements present (86). Minerals are micronutrients needed in minute quantities, which cannot be produced by the body but are essential for good health.

The total carbohydrate content of the *ogi* powder ranged from 77.47to 79.51%, and they were significantly different (*p* < 0.05) from one another. The control (Maize 4) recorded the highest carbohydrate content (79.51%) while Maize 2 had the lowest (77.47%). This result is comparable to that of previous studies (17, 81–83). The differences observed among the *ogi* powder samples can be due to other nutrients and the breakdown of starch used as a source of the substrate by the microorganism during fermentation in the steeping unit.

### Contribution of *Ogi* to Daily Vitamin A Intake

The possible contribution of *ogi* to vitamin A intake was calculated based on the assumption that the popular white-colored *ogi* is replaced with *ogi* from the new biofortified PVA genotypes of maize. The result is presented in Table 5. The *ogi* powder is usually prepared into a porridge/pap/maize gruel popularly consumed and sweetened with sugar or milk. One serving of maize pap was reported by Sanusi and Olurin (60) as weighing 219.3 g, and this was applied as a benchmark for calculating the contribution to vitamin A intake for all age groups. The same study established that the mean portion size usually consumed by an adult was about two and a half servings. From these extrapolations, Maize 2 contributed the highest to vitamin A intake across all age groups while Maize 1 contributed the lowest. The highest possible contribution of one serving to nutrient intake was found in children aged 1–8 years at 18.3% of the estimated average requirement (EAR).

**Table 5:** Percentage contribution of one serving of *ogi* pap/porridge (219 g) to estimated average requirement (EAR) of vitamin A.

**Age group (years)**	**EAR (μg)**	**% contribution of serving of *ogi***
Children 1–8 yrs	210–275	MAIZE 1 (10.2–13.4)
		MAIZE 2 (14.0–18.3)
		MAIZE 3 (13.0–17.0)
		MAIZE 4 (13.1–17.2)
Children 9–13 yrs	420–445	MAIZE 1 (6.7–6.3)
		MAIZE 2 (8.6–9.2)
		MAIZE 3 (8.0–8.5)
		MAIZE 4 (8.1–8.6)
Adolescents female 14–18 yrs	485	MAIZE 1 (5.8)
		MAIZE 2 (7.9)
		MAIZE 3 (7.3)
		MAIZE 4 (7.4)
Adolescents male 14–18 yrs	630	MAIZE 1 (4.5)
		MAIZE 2 (6.1)
		MAIZE 3 (5.7)
		MAIZE 4 (5.7)
Adult females >19 yrs	500	MAIZE 1 (5.6)
		MAIZE 2 (7.7)
		MAIZE 3 (7.1)
		MAIZE 4 (7.2)
Adult males >19 yrs	625	MAIZE 1 (4.5)
		MAIZE 2 (6.2)
		MAIZE 3 (5.7)
		MAIZE 4 (5.8)

Furthermore, the result shows a decrease in the contribution of one serving as the age increased, which implies that the older the person, the more the portion needed. During processing, about three-fourths of the provitamin-A content was lost; this has reduced the potential of *ogi* to be a rich PVA source. Assuming more than one serving is consumed, an adult will have to consume more than the reported average portion of 526 g to meet up the expectation of 50% EAR (33, 87). These low estimations are similar to the reports on cassava products presented by Eyinla et al. (88), who reported an established negative impact of processing on the retention and contribution to vitamin A intake caused by processing. The variance in contribution to EAR within the genotype was not large, which implies that, despite a significant difference in the composition of PVA, potency and contribution will be low.

### Phytate Content in *Ogi* Powder

Table 6 presents the phytate content before (maize flour) and after processing (*ogi* powder) and shows significant differences at *p* < 0.05. The phytate content in maize kernel before production into *ogi* powder ranged from 4.09 to 5.96 mg/g; the highest was in Maize 1 and the lowest in Maize 2. After production into *ogi* powder, the phytate content decreased to the range of 1.70–2.78 mg/g in the maize samples. The lowest was observed in the control variety (Maize 4) while the highest was Maize 1. However, among the PVA maize, the lowest was in Maize 2. The decreased phytate content in the *ogi* powder was due to the fermentation process; organisms involved in steeping during fermentation cause the activation of hydrolysis of phytate by phytase (89, 90). Also, it was observed that the unit operations (Milling to form a slurry) and the duration (Steeping for 48 h) in Figure 1 aided phytate content reduction (91). This reduction is in accordance with the observations (21, 49, 92–94).

**Table 6:** Phytate content before and after production into *ogi* powder.

**Genotypes**	**^+^Before**	**After**
**Maize 1**	5.96 ± 0.03^c^	2.78 ± 0.07^d^
**Maize 2**	4.09 ± 0.03^a^	1.91 ± 0.07^b^
**Maize 3**	5.80 ± 0.06^b^	2.27 ± 0.10^c^
**Maize 4**	5.76 ± 0.03^b^	1.70 ± 0.07^a^

*Values are mean ± SD of triplet determination*.

+ *Values with different superscripts along the column are significantly different from one another (p ≥ 0.05)*.

### Sensory Evaluation of *Ogi* Porridge

Table 7 presents the sensory attributes of *ogi* porridge from biofortified PVA genotypes and landrace yellow maize. It shows significant differences (*p* < 0.05) among the *ogi* porridge in color, texture, and flavor while taste and overall acceptability are not significant.

**Table 7:** Sensory attributes of *ogi* porridge from provitamin A-biofortified maize genotypes and yellow landrace.

**Product**	**^+^Color**	**Taste**	**Texture**	**Flavor**	**Overall acceptability**
*Ogi* 1	6.78 ± 1.68^a^	6.45 ± 1.46^a^	6.50 ± 1.39^a^	6.23 ± 1.95^a^	6.40 ± 1.40^a^
*Ogi* 2	7.13 ± 1.26^ab^	7.03 ± 1.19^a^	7.20 ± 1.39^a^	6.78 ± 1.53^a^	7.25 ± 1.21^b^
*Ogi*3	7.73 ± 1.23^b^	6.95 ± 1.62^a^	7.15 ± 1.49^a^	7.18 ± 1.26^a^	7.40 ± 1.22^b^
*Ogi* 4 (control)	7.25 ± 1.13^ab^	6.90 ± 1.26^a^	6.68 ± 1.31^a^	6.60 ± 1.41^a^	6.86 ± 1.09^ab^

*Values are mean ± SD of triplet determination*.

+ *Values with different superscript along the column are significantly different from one another (p ≥ 0.05)*.

Color parameters of the *ogi* porridge samples ranged from 6.78 to 7.73, and the color of *ogi* 3 porridge is the most preferred. Table 7 shows the color of the *ogi* porridge that has been accepted mainly among the panelists. Color is an attractant property of food that is usually attributed to wellness and quality. The results revealed that *ogi* 1–4 were not significantly different in taste (*p* > 0.05). This signifies that *ogi* porridges from biofortified PVA maize genotypes and the yellow landrace have a similar tart sourness taste, an essential factor in *ogi* porridge caused by the fermentation of the carbohydrates by microorganisms such as lactic acid bacteria (*Lactobacillus plantarum* and *Streptococcus lactis*) and yeasts (*Saccharomyces cerevisiae* and *Debarmyces hansenii*) (10, 16). The texture is a very important factor for the consumption of *ogi* porridge. The smooth texture of *ogi* porridge varies due to perception. The texture results presented in Table 7 show that *ogi* 2 and 3 were liked moderately while *ogi* 1 and 4 were liked slightly. The ratings for flavor showed significant differences at *p* < 0.05. The *ogi* porridge (*ogi* 3) from PVA SYN HGBC_1_ ranked the best in terms of texture, color, flavor, and overall acceptability with the mean values of 7.15, 7.73, 7.18, and 7.40, respectively, in comparison with the yellow maize and all other biofortified PVA maize genotypes. Nevertheless, all the other biofortified PVA maize genotypes with yellow maize were moderately liked and accepted.

## Conclusion

This study evaluated the chemical composition, sensory perception, phytate content, retention, and the possible contribution of PVA in newly bred genotypes of biofortified maize. The composition of different carotenoids varied between the provitamin-A genotypes pre-and post-processing, with Maize 2 retaining the highest post-processing. The reduction in the composition of PVA retention was affected by the unit operations involved in the traditional processing of *ogi* powder. Also, the highest possible contribution of vitamin A intake across all age groups was observed in Maize 2 (PVA SYN HGBC_0_). Maize 2 had the lowest phytate content among the PVA maize before and after processing into *ogi* powder, and *ogi* 3 porridge (PVA SYN HGBC_1_) was the most acceptable one. From this study, biofortification and retention estimation can be used as a tool to produce nutritious foods to achieve Zero Hunger (SDG 2). PVA genotypes, such as PVA SYN HGBC0, with a higher quantity of carotenoids to combat VAD, can still be worked on in the ongoing maize improvement program.

## Data Availability Statement

The raw data supporting the conclusions of this article will be made available by the authors, without undue reservation.

## Author Contributions

EA and BO: conceptualization and supervision. EA and BM-D: methodology and funding acquisition. BO and OA: software. EA, BO, BM-D, and AM: validation and writing—review and editing. OA, EA, and BO: formal analysis, investigation, and data curation. BM-D and AM: resources. OA: writing—original draft preparation. All authors have read and agreed to the published version of the manuscript.

## Conflict of Interest

The authors declare that the research was conducted in the absence of any commercial or financial relationships that could be construed as a potential conflict of interest.
